# Supplementary figures and images for: C1-FDX is required for the assembly of mitochondrial complex I and subcomplexes of complex V in Arabidopsis
Source: PLoS Genet. 2024 Oct 2;20(10):e1011419. doi: 10.1371/journal.pgen.1011419 (PMC11446459; doi:10.1371/journal.pgen.1011419)

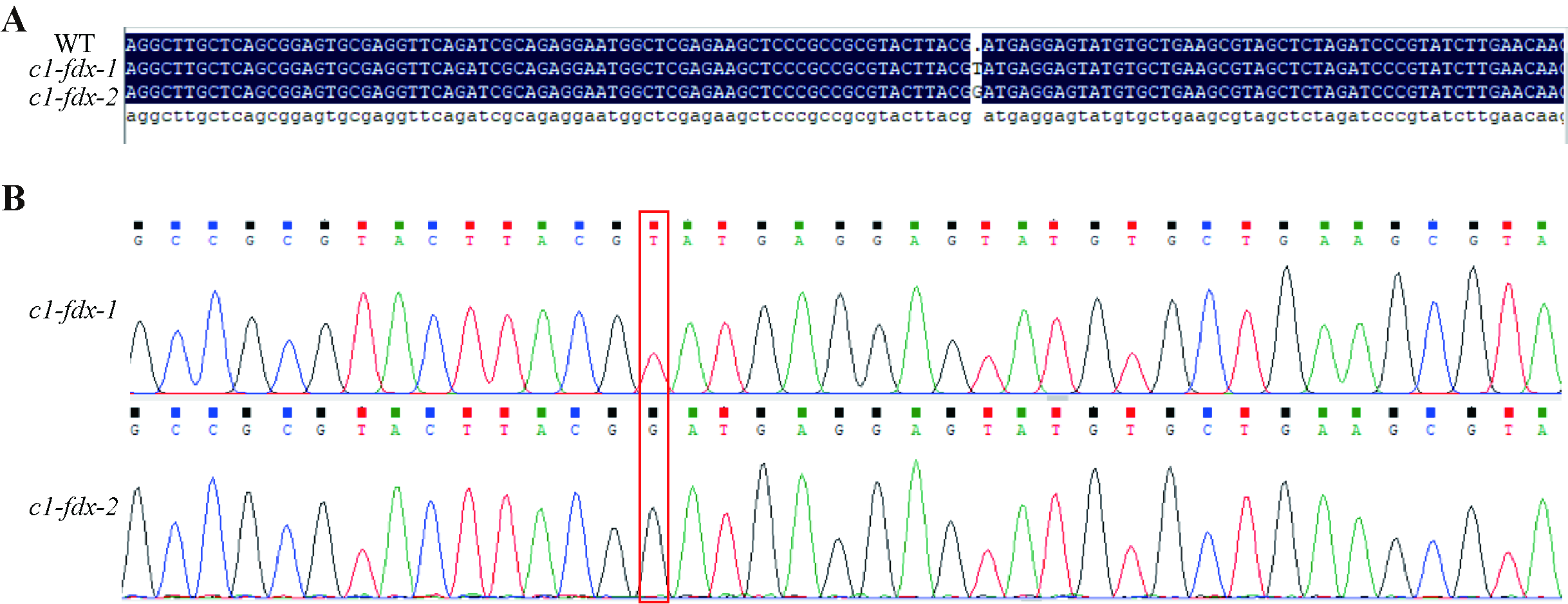

Supplement: S1 Fig — (TIF) [file pgen.1011419.s001.tif]

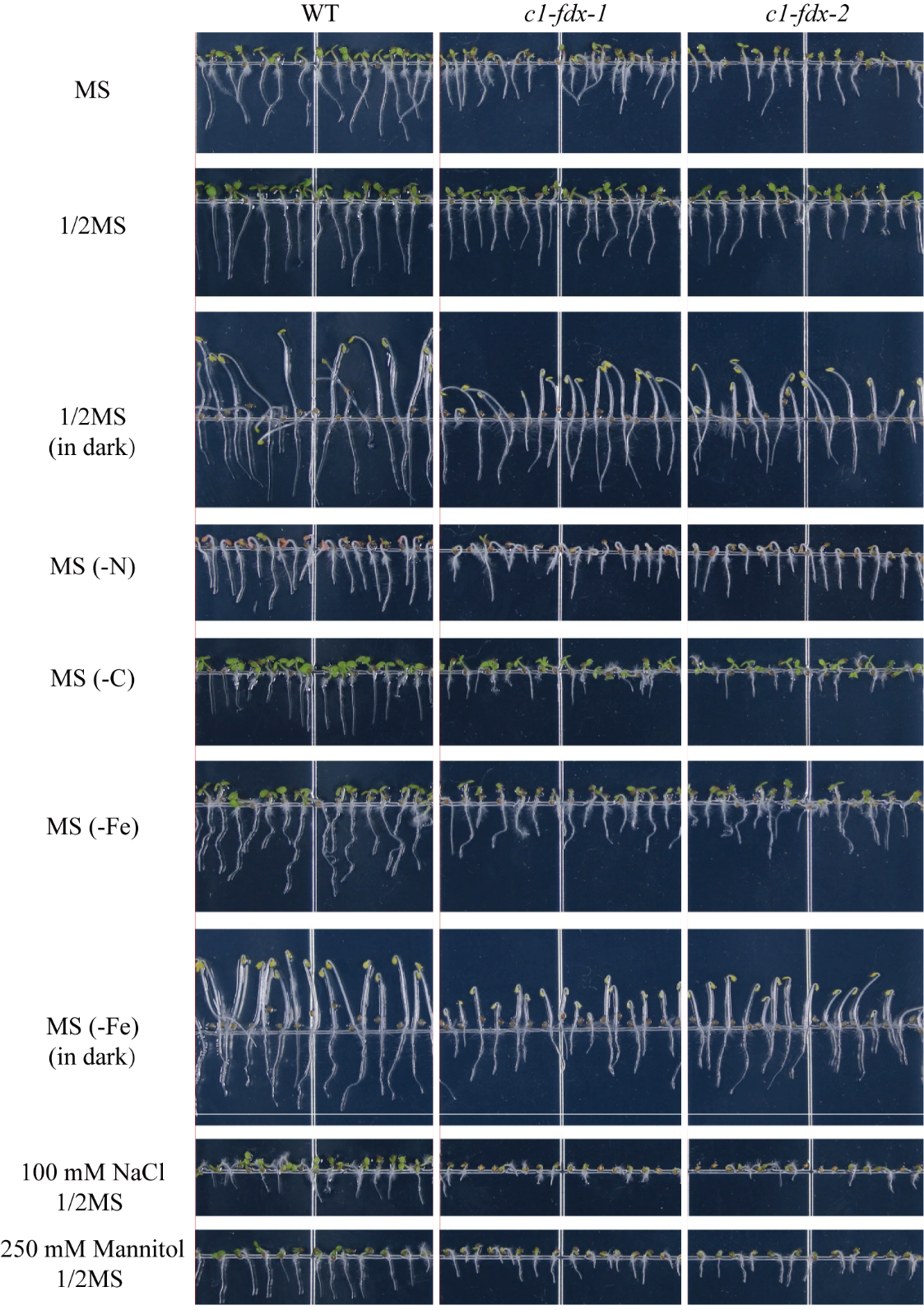

Supplement: S2 Fig — (TIF) [file pgen.1011419.s002.tif]

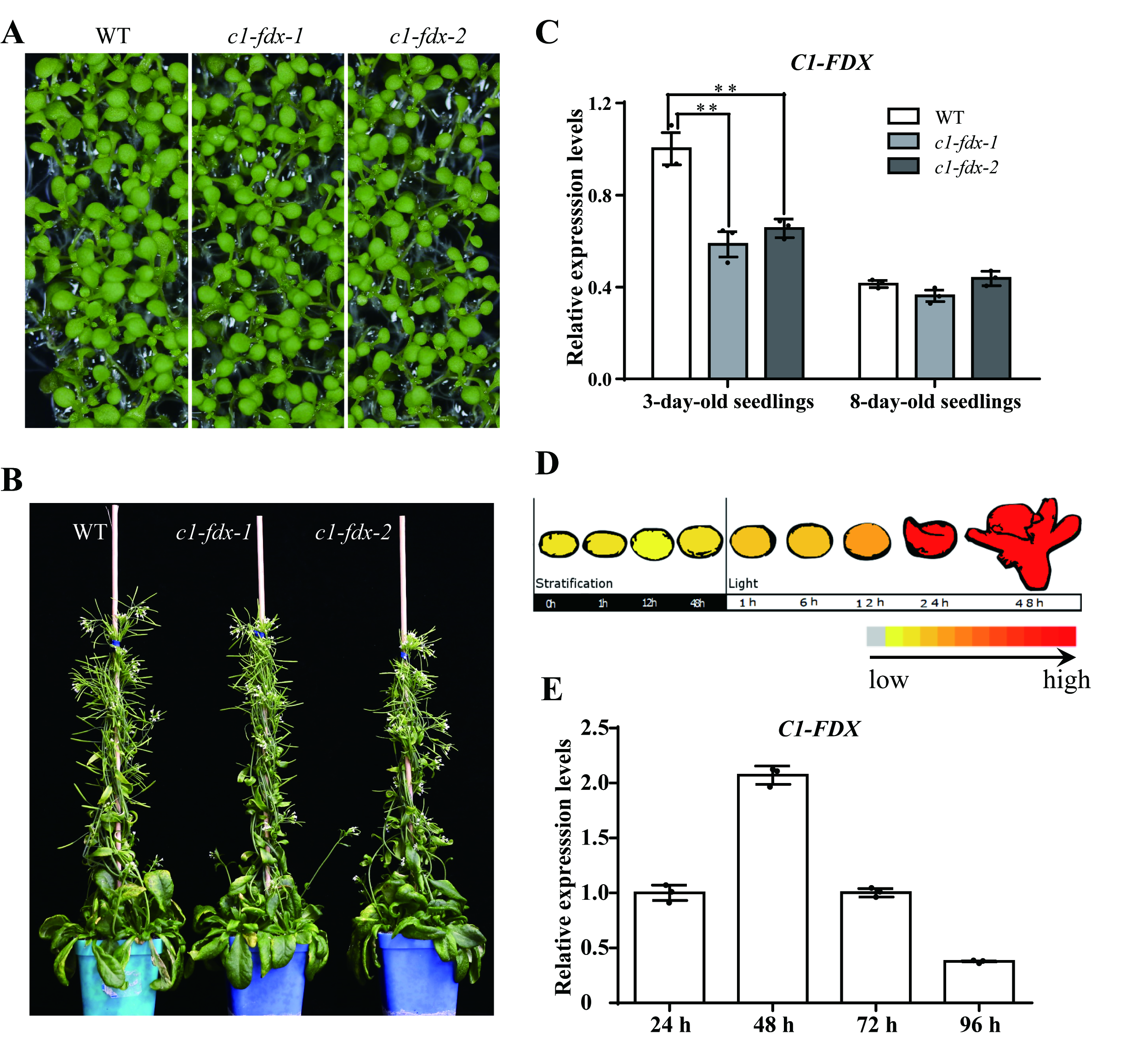

Supplement: S3 Fig — (TIF) [file pgen.1011419.s003.tif]

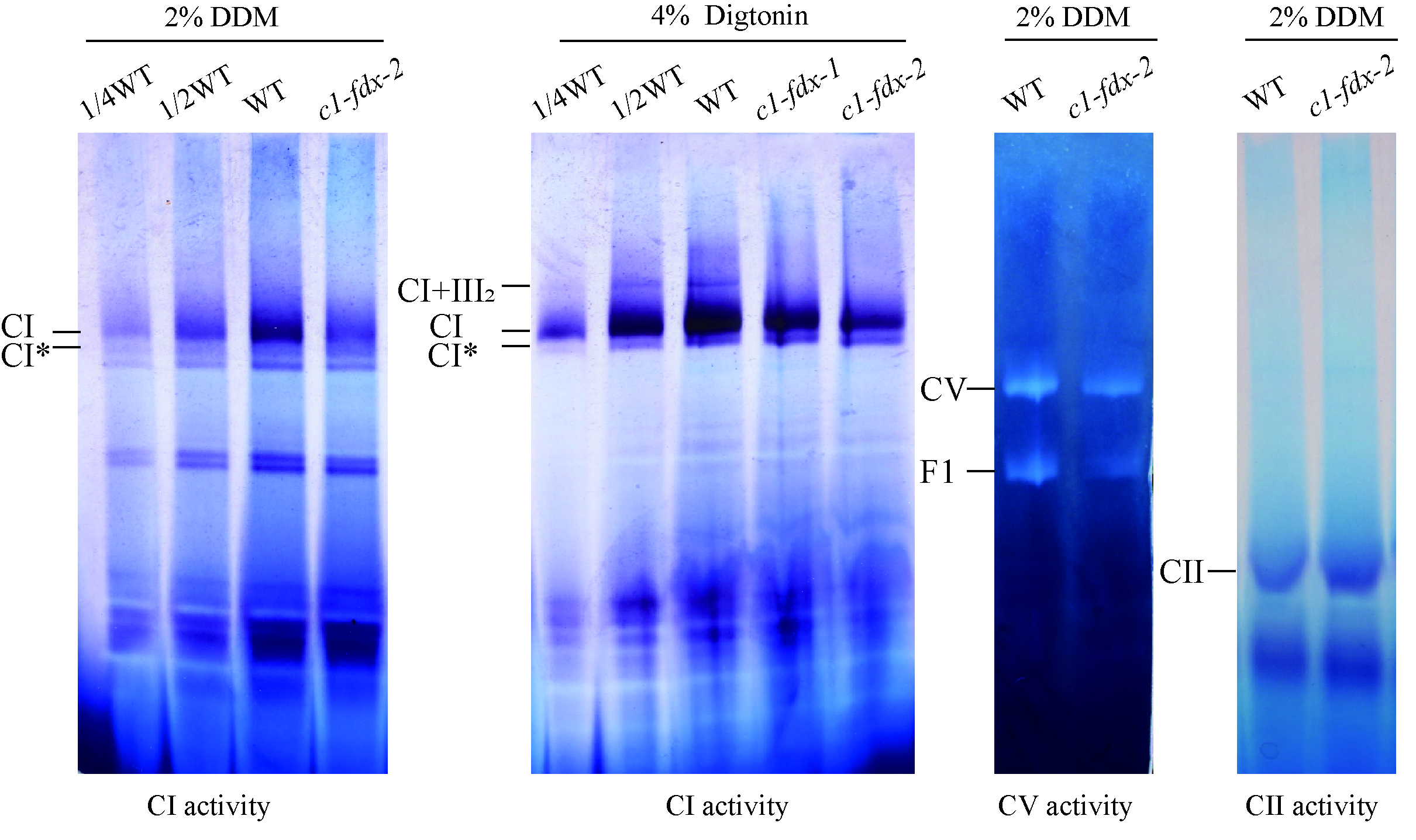

Supplement: S4 Fig — (TIF) [file pgen.1011419.s004.tif]

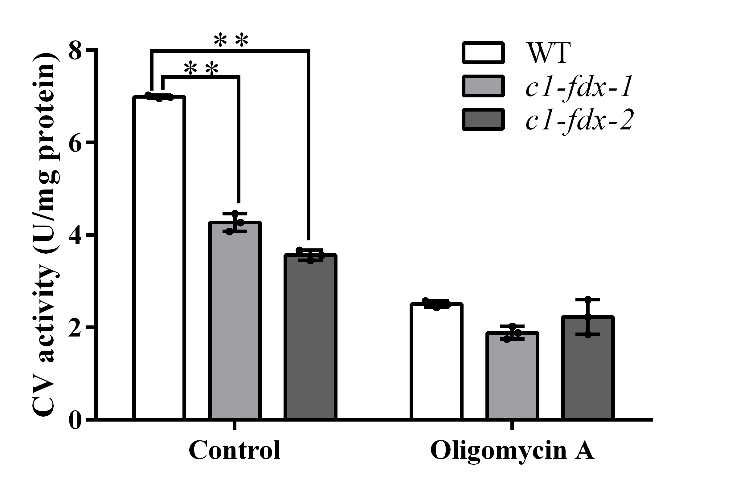

Supplement: S5 Fig — (TIF) [file pgen.1011419.s005.tif]

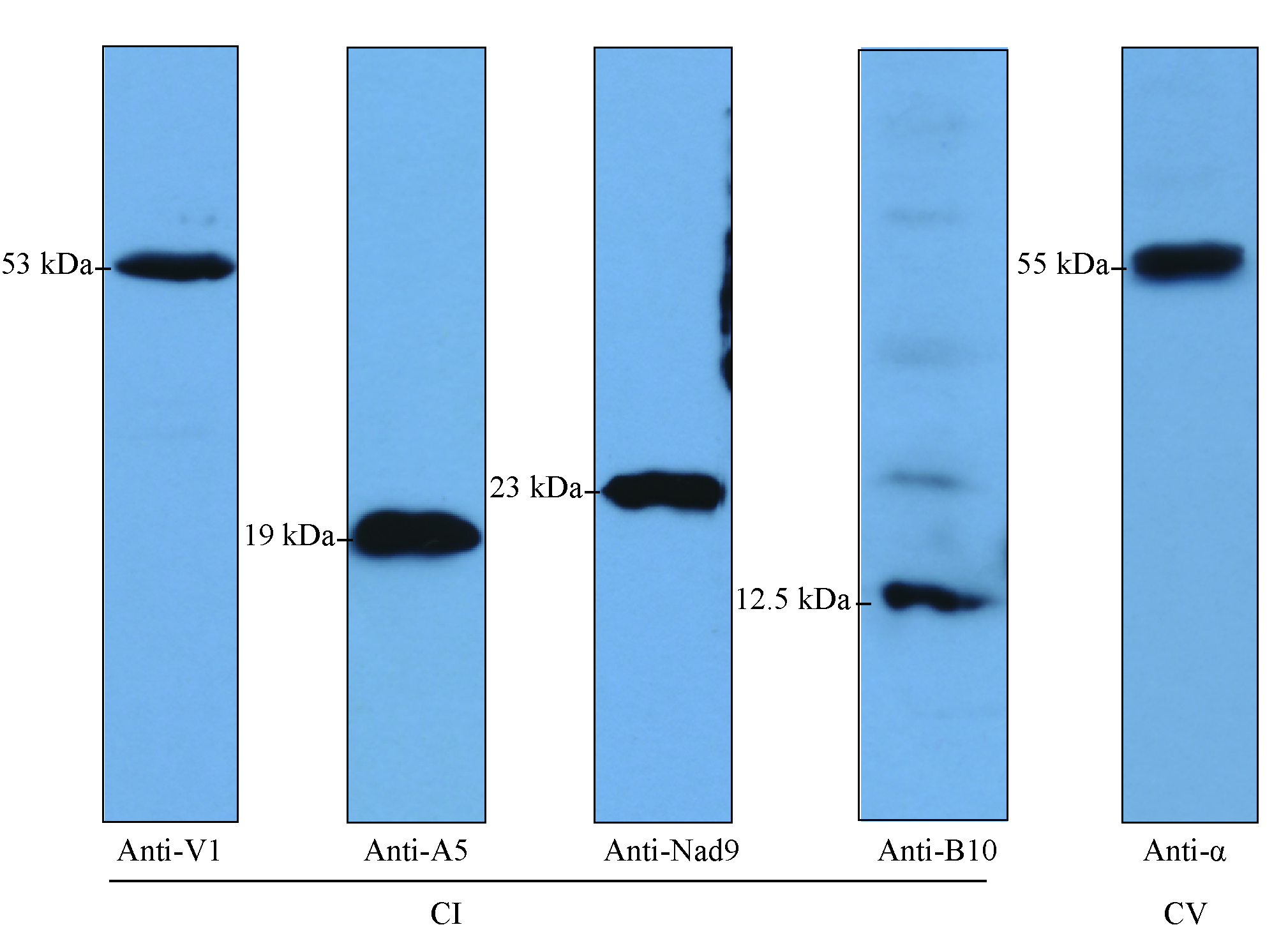

Supplement: S6 Fig — (TIF) [file pgen.1011419.s006.tif]

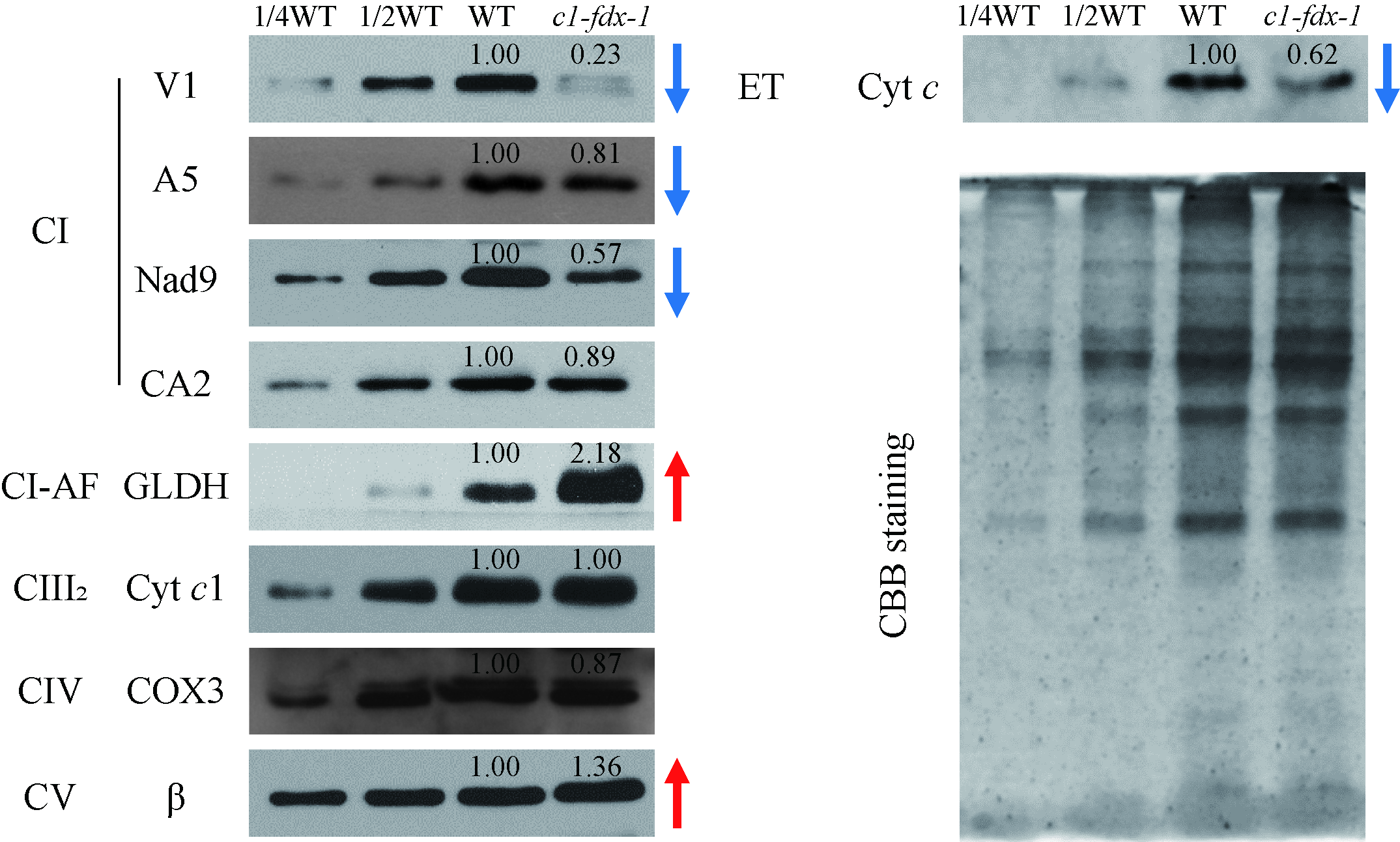

Supplement: S7 Fig — (TIF) [file pgen.1011419.s007.tif]

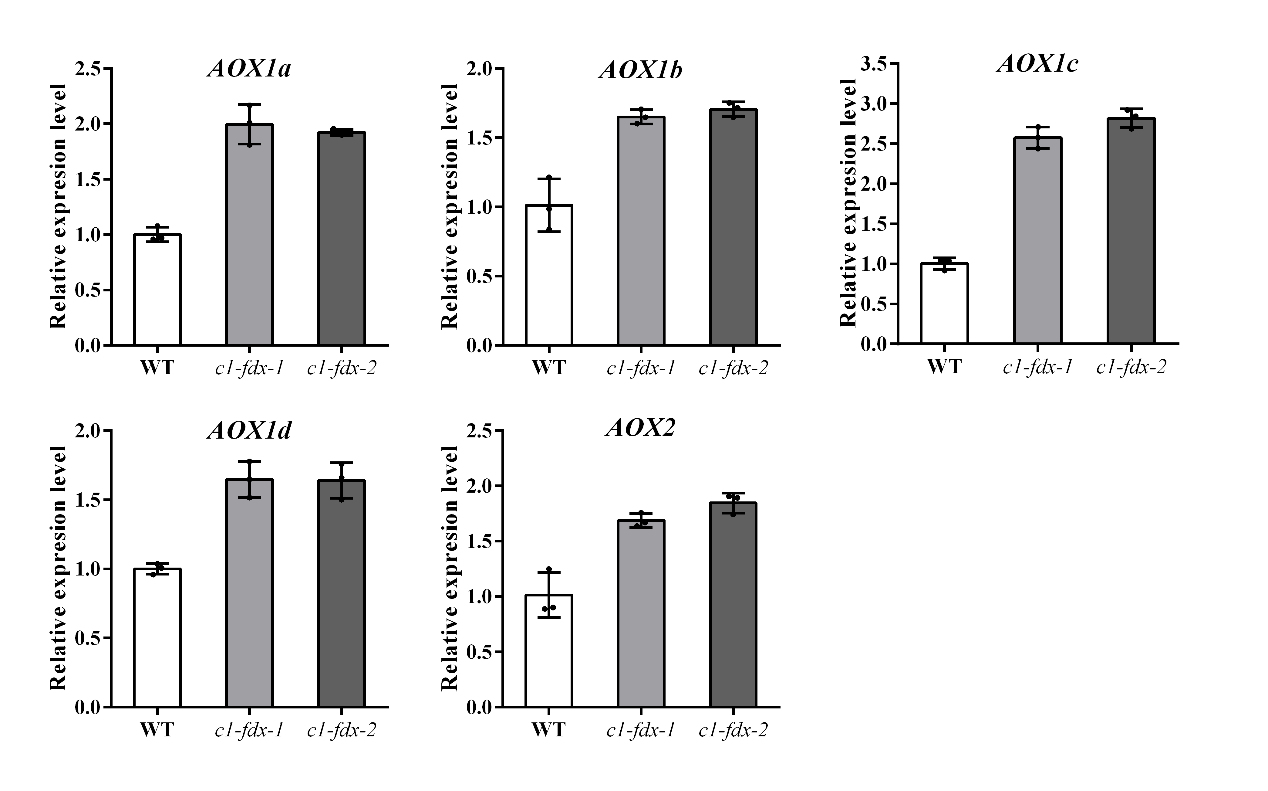

Supplement: S8 Fig — (TIF) [file pgen.1011419.s008.tif]

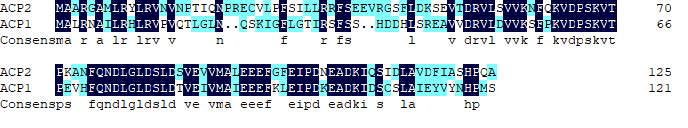

Supplement: S9 Fig — (TIF) [file pgen.1011419.s009.tif]

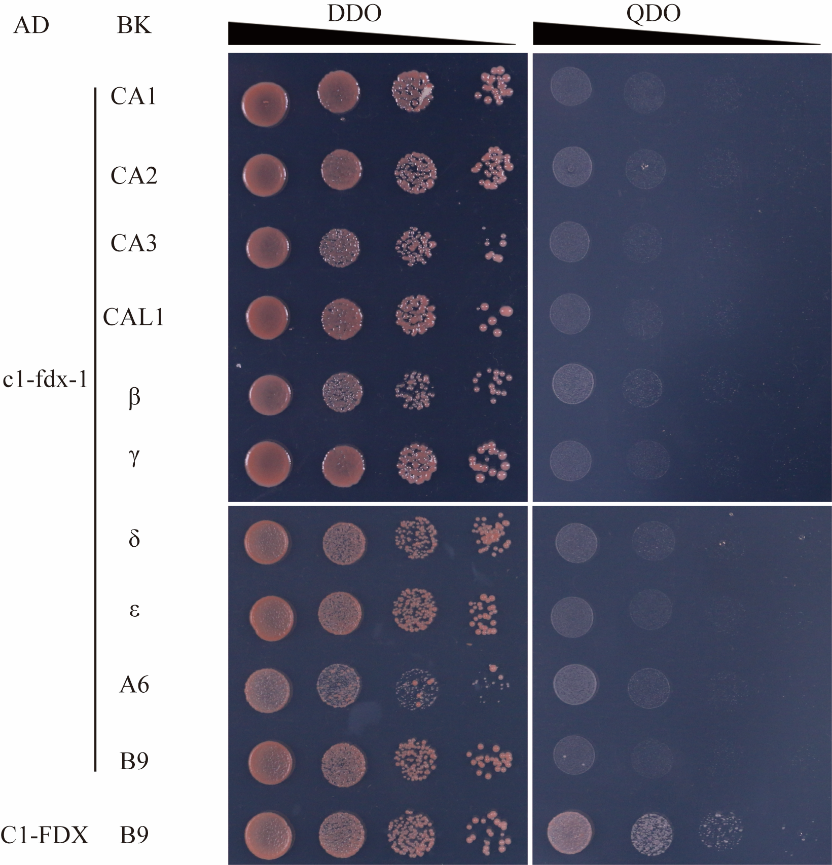

Supplement: S10 Fig — (TIF) [file pgen.1011419.s010.tif]

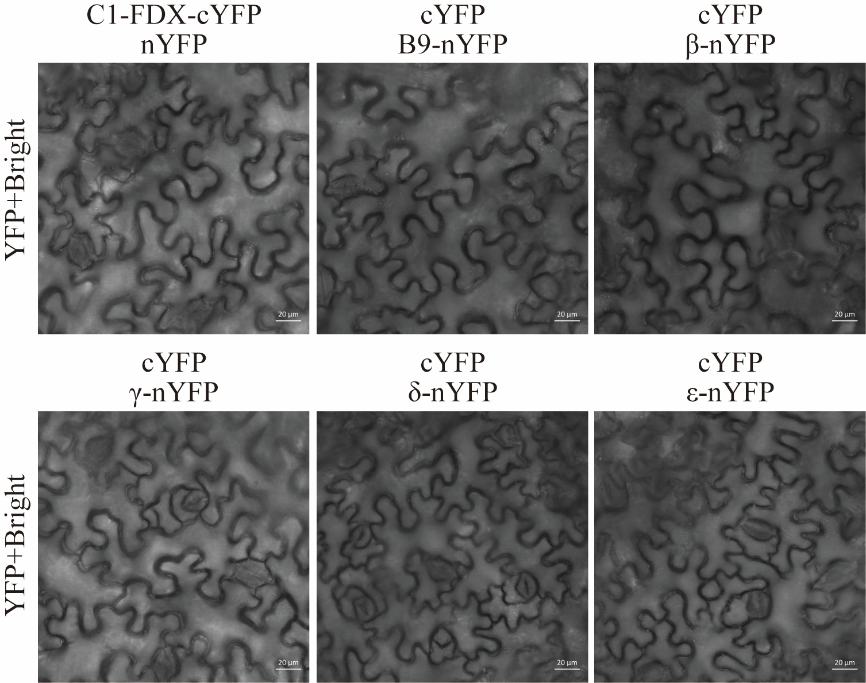

Supplement: S11 Fig — (TIF) [file pgen.1011419.s011.tif]

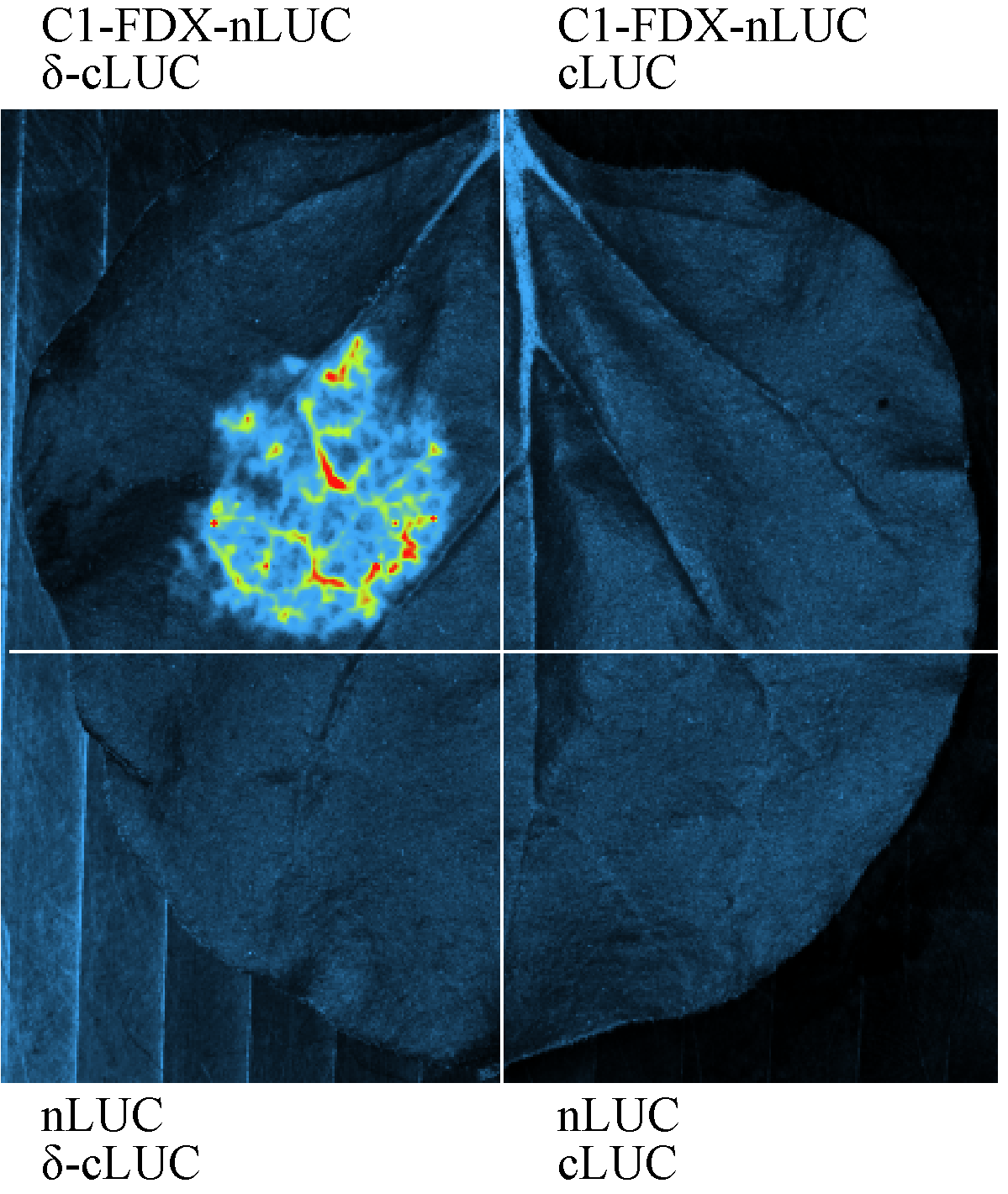

Supplement: S12 Fig — (TIF) [file pgen.1011419.s012.tif]
