# Supplementary material for: C1-FDX is required for the assembly of mitochondrial complex I and subcomplexes of complex V in Arabidopsis
Source: PLoS Genet. 2024 Oct 2;20(10):e1011419. doi: 10.1371/journal.pgen.1011419 (PMC11446459; doi:10.1371/journal.pgen.1011419)
Supplement: S2 Table — (DOCX) [file pgen.1011419.s014.docx]

**S2 Table. Primers were used in this study.**

| **Primer name** | **Primer sequence (5’ to 3’)** | **Use for** |
| --- | --- | --- |
| *C1-FDX-F1* | TCAACCCAAACTAAACCAAACA | Genotyping primer |
| *C1-FDX-R1* | TCAAGAACAAAAACTTACGGAA |  |
| *C1-FDX-F2* | CACCATGGCGACGACTCTTCAGAA | Construct pGWB5-C1-FDX vector |
| *C1-FDX-R2* | CGGAATATCCCAAGGCTTAG |  |
| *C1-FDX-F3* | AACTGCAGATGGCGACGACTCTTCAGAA | Construct pSuper1300-C1-FDX vector without GFP sequence |
| *C1-FDX-R3* | CGAGCTCTTACGGAATATCCCAAGGCTT |  |
| DT1-BsF | ATATATGGTCTCGATTGCCGCCGCGTACTTACGATGGTT | Mutation of *C1-FDX* gDNA by CRISPR/Cas9 system |
| DT1-F0 | TGCCGCCGCGTACTTACGATGGTTTTAGAGCTAGAAATAGC |  |
| DT2-R0 | AACAGACGCGAGTGCTTGTTCACAATCTCTTAGTCGACTCTAC |  |
| DT2-BsR | ATTATTGGTCTCGAAACAGACGCGAGTGCTTGTTCACAA |  |
| *AOX1a-F* | GTTTCGTCTCACGAGGCTTTAT | Gene expression |
| *AOX1a-R* | GGTGGATTCGTTCTCTGTTTTC |  |
| *AOX1b-F* | CAAGCTAATGGAAACTGCTGTG |  |
| *AOX1b-R* | CATCTTGCTGAAAACTCTCACG |  |
| *AOX1c-F* | ATTACTCCGTCGCTCTCTCCTT |  |
| *AOX1c-R* | CTTCACGCCCCAATAACTAACT |  |
| *AOX1d-F* | GGATTCAGGGGACATCTCATTA |  |
| *AOX1d-R* | CTGGCTGGTTATTCCCACTTAC |  |
| *AOX2-F* | CATGTTCGTGAGTTCTGTTTCC |  |
| *AOX2-R* | ACCCATCCACCTCAAGTTAAAA |  |
| *AtActin1-F* | GGTAACATTGTGCTCAGTGGTG |  |
| *AtActin1-R* | GCAGCATGAAGATCAAAGTCG |  |
| *C1-FDX-F4* | ATGGCCATGGAGGCCGAATTCATGGCGACGACTCTTCAGAAA | Construct Y2H vectors |
| *C1-FDX-R4* | CTAGTTATGCGGCCGCTGCAGTTACGGAATATCCCAAGGCTTAG |  |
| *B9-F1* | ATGGCCATGGAGGCCAGTGAATTCATGAGCGGAGTTTCGACGG |  |
| *B9-R1* | GCAGCTCGAGCTCGATGGATTCTTATGGGTTGTCTTCTAGACCATAGTT |  |
| *β-F1* | ATGGCCATGGAGGCCGAATTCATGGCGTCTCGGAGAGTCTTAT |  |
| *β-R1* | CTAGTTATGCGGCCGCTGCAGTTAAGCTGCTGACTCTTTAGCGA |  |
| *γ-F1* | ATGGCCATGGAGGCCGAATTCATGGCAATGGCTGTTTTCC |  |
| *γ-R1* | CTAGTTATGCGGCCGCTGCAGTTATTTAGCAGCTTCAAGAGCAGA |  |
| *δ-F1* | ATGGCCATGGAGGCCGAATTCATGTTTAAACAAGCTTCTCGTCTC |  |
| *δ-R1* | CTAGTTATGCGGCCGCTGCAGTTAGCCCGAGAGAGCTGC |  |
| *ε-F1* | ATGGCCATGGAGGCCGAATTCATGGCATCGAATGCGGC |  |
| *ε-R1* | CTAGTTATGCGGCCGCTGCAGTCAAACTTCAGGTGTGTCTGACC |  |
| *A6-F1* | ATGGCCATGGAGGCCAGTGAATTCATGGCGGCACCATTCG |  |
| *A6-R1* | GCAGCTCGAGCTCGATGGATTCTCAAAAGTAGTTGCTGGTGTAGAAA |  |
| *OSCP-F1* | ATGGCCATGGAGGCCAGTGAATTCATGGCTAATCGTTTCAGATCAGG |  |
| *OSCP-R1* | GCAGCTCGAGCTCGATGGATTCCAACAGGTTCACGGAGGAGC |  |
| *c-F1* | ATGGCCATGGAGGCCAGTGAATTCATGACAAAGCGTGAGTATAATTCTCA |  |
| *c-R1* | GCAGCTCGAGCTCGATGGATTCTCAGAATACGAATAAGATCAAAAAGG |  |
| *d-F1* | ATGGCCATGGAGGCCAGTGAATTCATGAGCGGAGCCGGTAAGA |  |
| *d-R1* | GCAGCTCGAGCTCGATGGATTCTCAGTATCCCCAGTTGTCATTACG |  |
| *a-F1* | ATGGCCATGGAGGCCAGTGAATTCATGCGACGAATCTTTTTGTTTG |  |
| *a-R1* | GCAGCTCGAGCTCGATGGATTCTTAATGGAGATTTATAGCATCATTCAA |  |
| *ACP1-F1* | GTACCAGATTACGCTCATATGATGGCACTGAGAAATGCAATT |  |
| *ACP1-R1* | GCAGCTCGAGCTCGATGGATTCTTAGCTAGACATTGGATGATTGTAAA |  |
| *ACP2-F1* | ATGGCCATGGAGGCCAGTGAATTCATGGCGGCGAGAGGTG |  |
| *ACP2-R1* | GCAGCTCGAGCTCGATGGATTCTCACTTAGCCTGAGGATGAGAAG |  |
| *α-F1* | ATGGCCATGGAGGCCGAATTCATGATTACTCGGCTGTTCGC |  |
| *α-R1* | CTAGTTATGCGGCCGCTGCAGCTAAATTAAAGCTAAAGCTCTTTCTTTTA |  |
| *GLDH-F1* | ATGGCCATGGAGGCCAGTGAATTCATGCTCCGGTCACTTCTTCTC |  |
| *GLDH-R1* | GCAGCTCGAGCTCGATGGATTCTTAAGCAGTGGTGGAGACTGG |  |
| *C1-FDX-F5* | CGCGGATCCATGGCGACGACTCTTCAGAA | Construct BiFC vectors |
| *C1-FDX-R5* | TCCCCCGGGCGGAATATCCCAAGGCTTAG |  |
| *B9-F2* | CGCGGATCCATGAGCGGAGTTTCGACG |  |
| *B9-R2* | CCGCTCGAGTGGGTTGTCTTCTAGACCATAGTT |  |
| *β-F2* | GCTCTAGAATGGCGTCTCGGAGAGTCT |  |
| *β-R2* | GGACTAGTAGCTGCTGACTCTTTAGCGATC |  |
| *γ-F2* | GCTCTAGAATGGCAATGGCTGTTTTCC |  |
| *γ-R2* | CCGCTCGAGTTTAGCAGCTTCAAGAGCAGAA |  |
| *δ-F2* | GCTCTAGAATGTTTAAACAAGCTTCTCGTCTC |  |
| *δ-R2* | CCGCTCGAGGCCCGAGAGAGCTGCG |  |
| *ε-F2* | GCTCTAGAATGGCATCGAATGCGG |  |
| *ε-R2* | CCGCTCGAGAACTTCAGGTGTGTCTGACCG |  |
| *C1-FDX-F6* | AACTGCAGATGGCGACGACTCTTCAGAA | Construct Co-IP vectors |
| *C1-FDX-R6* | CGGGGTACCCGGAATATCCCAAGGCTTAG |  |
| *B9-F3* | AACTGCAGATGAGCGGAGTTTCGACG |  |
| *B9-R3* | CGGGGTACCTGGGTTGTCTTCTAGACCATAGTT |  |
| *β-F3* | GCTCTAGAATGGCGTCTCGGAGAGTCT |  |
| *β-R3* | CGGGGTACCAGCTGCTGACTCTTTAGCGATC |  |
| *γ-F3* | GCTCTAGAATGGCAATGGCTGTTTTCC |  |
| *γ-R3* | CGGGGTACCTTTAGCAGCTTCAAGAGCAGAA |  |
| *ε-F3* | GCTCTAGAATGGCATCGAATGCGG |  |
| *ε-R3* | CGGGGTACCAACTTCAGGTGTGTCTGACCG |  |
| *C1-FDX-F7* | CGAGCTCATGGCGACGACTCTTCAGAA | Construct LCI vectors |
| *C1-FDX-R7* | CGGGGTACCCGGAATATCCCAAGGCTTAG |  |
| *δ-F3* | CGAGCTCATGTTTAAACAAGCTTCTCGTCTC |  |
| *δ-R3* | CGAGCTCGCCCGAGAGAGCTGCG |  |
