# Supplementary material for: C1-FDX is required for the assembly of mitochondrial complex I and subcomplexes of complex V in Arabidopsis
Source: PLoS Genet. 2024 Oct 2;20(10):e1011419. doi: 10.1371/journal.pgen.1011419 (PMC11446459; doi:10.1371/journal.pgen.1011419)
Supplement: Attachment — Submitted filename: Response to Reviewers_C1-FDX_PLOS genetics.docx [file pgen.1011419.s015.docx]

**Reviewer #1:**

The manuscript entitled “C1-FDX is required for the assembly of mitochondrial complexes I and V in Arabidopsis” presents an interesting investigation into the physiological function of the so-called Complex I ferredoxin (C1-FDX) by Baoyin Chen et al. The authors assert that C1-FDX serves as an essential assembly factor for mitochondrial complexes I and V. The manuscript is well-written, and the conclusions are supported by the experiments; however, I have some comments.

1-In line 65, the authors state, “In humans, mFDX1 and mFDX2 play an essential role in the assembly of the Fe-S cluster and the biosynthesis of heme A (Sheftel et al., 2010). However, the function of mFDX1 and mFDX2 in plants remains to be revealed.”
This statement may not be entirely accurate, as there are relevant papers that should be cited. For instance, the plant ADX–ADXR system has been linked to biotin synthesis in collaboration with BIO2, an iron–sulfur (Fe-S) cluster enzyme (Picciocchi et al., 2003). Additionally, Bellido AM et al. (2022) identified a mitochondrial ADXR–ADX–P450 electron transport chain crucial for maternal gametophytic control of embryogenesis in Arabidopsis. This chain, involving mFDXs (ADX1 and ADX2), is associated with steroid hormone synthesis.

Response: Thanks for your suggestion. We have cited the papers and revised the description in Lines 76-80: “In humans, mFDX1 and mFDX2 play an essential role in the assembly of the Fe-S cluster and the biosynthesis of heme A (Sheftel et al., 2010). In Arabidopsis, mFDX1 and mFDX2 are crucial for maternal gametophytic control of embryogenesis, which is associated with steroid hormone synthesis and biotin synthesis (Picciocchi et al., 2003; Bellido et al., 2022).”

2-The manuscript does not clearly specify the expected peptide or truncated C1-FDX. Including this information in Figure 1 or discussing the possibility of detecting this peptide via LC:MS/MS would be beneficial. It is also essential to consider potential interactions and functionality of the truncated peptide, which comprises a substantial portion of the wildtype protein.

Response: Thanks for the suggestion. We added the information in the text between lines 135-139 and Figure 1. The single base “T” or “G” insertion at +338bp in the coding region of C1-FDX causes a frameshift, which generates a TGA stop codon immediately after the insertion (Figure 1A and B). This mutation truncates the 46 amino acid sequence of the C1-FDX C-terminus if it is translated. C1-FDX is 159 Aa residues, i.e., truncating 29% of the protein. This information is added to the revised manuscript. The truncated c1-fdx protein is probably not functional, at least not fully functional. We performed the LC-MS/MS analysis again on the F’ subcomplex in the wildtype, c1-fdx-1 mutant, and complemented line. C1-FDX peptides were detected in the wildtype and the complemented line, but again, not in the c1-fdx-1 mutant (Table S1), suggesting that the truncated c1-fdx loses the capability to incorporate into the F’ complex. Consistent with this result, the Y2H assay showed that the truncated c1-fdx-1 could not interact with the proteins that interact with the full-length C1-FDX (Figure 5 and S10). This information was added in the results (line 243-246 and 275-278).

3-Regarding BN gels, it is noted that the authors were unable to observe the supercomplex I + III2. C1-FDX may impact the stability of this supercomplex. I read in the M & M section that the authors use dodecylmaltoside or digitonin. In my experience, the supercomplex is only seen using digitonin but the gels that the authors show were solubilized with dodecylmaltoside. Have the authors seen any differences in complexes using digitonin?

Response: Yes, we observed the difference in the supercomplexes between using dodecylmaltoside and digitonin. The supercomplexes (I+III_2_) can be solubilized by digitonin but not by dodecylmaltoside. For this reason, we used digitonin as the detergent to solubilize the supercomplexes I+III_2_ from mitochondria of wildtype and *c1-fdx-1* in Figure S4. The result showed that the C1-FDX affects the assembly of the supercomplexes (I+III_2_) in Arabidopsis.

4-The manuscript mentions that overexpressed C1-FDX-GFP leads to increased amounts of complex I and activity. An explanation for this phenomenon should be provided.

Response: Thanks for your suggestion. We have added the explanation in the discussion in Lines 300-301. “This result implies that C1-FDX, as a component of the ferredoxin bridge protein, could promote formation of the ferredoxin bridge and then increase the assembly of CI*.” It is possible that the in vivo amount of C1-FDX is not limited; thus, an increased amount of C1-FDX-GFP leads to increased CI assembly of CI, or C1-FDX-GFP somehow stabilizes CI while still retaining function, or it could be through affecting complex V, which up-regulates the CI amount. These are speculations with any data to support them.

5-Concerning interaction experiments, the lack of interaction of CAL2 with any other protein raises questions. Clarification on the physiological significance of this observation is warranted, particularly considering the interchangeability of CAL1 and CAL2 in CI models.

Response: Genetic analysis shows that cal1 and cal2 single mutants do not display a significant impact on CI assembly. CAL1 and CAL2 share an 89% amino sequence identity, suggesting that both proteins are functional in a redundant manner. However, the Y2H analysis showed that CAL1 behaved differently from CAL2. We did not detect a similar interaction with CAL2. Maybe it is due to the yeast system or the different properties between CAL1 and CAL2. For example, CAL2 may need a bridge protein to interact with the subunits that can directly interact with CAL1. The two proteins have 11% sequence divergence. Because we are not aware of the function of CAL1 or CAL2 in CI, we can only speculate on its significance.

6-The interaction between both ACP1 and ACP2 with CAL1 and not CAL2 requires an explanation of its physiological relevance.

Response: Same as the above question. The results showed a difference in the interactions between CAL1 and CAL2 with other proteins. Because we are not aware of the function of CAL1 or CAL2 in CI, we cannot offer much on its physiological relevance.

7-BIFC experiments are deemed unconvincing, and troubleshooting with protocols is suggested to address the issue of observing only a few concentrated yellow dots in a particular zone.
Response: According to your suggestion, we added the negative controls between C1-FDX and B9, β, γ, δ, ε in the BiFC analysis in Figure S11. None of these proteins can be self-activated in the BiFC assay.

8-While immunoprecipitations are clear, it is unclear why the interactions between C1-FDX and the CA proteins observed by Y2H were not confirmed.

Response: A recent study has shown that C1-FDX interacts with the CA domain (Klusch et al., 2021). For this reason, we did not test the interaction between C1-FDX and CA domain subunits by Co-IP.

Reference：

Klusch N, Senkler J, Yildiz Ö, Kühlbrandt W, Braun HP. A ferredoxin bridge connects the two arms of plant mitochondrial complex I. Plant Cell. 2021;33: 2072-2091.

9-Testing interactions with the truncated protein in addition to the full-length protein would add valuable insights and might explain some inconsistences.

Response: Thanks for the suggestion. We performed the experiment testing the interaction between truncated c1-fdx-1 protein and the C1-FDX interacting proteins. The result showed that the truncated c1-fdx-1 protein could not interact with any of these proteins (Figure S10), indicating that the truncated c1-fdx-1 protein lost its protein-protein interaction capability.

10-The manuscript raises the question of why, in a defective C1-FDX background plants, exhibit still reduced amounts of complexes I and V. Exploration of redundant proteins that may compensate for C1-FDX deficiency (if any) is suggested.

Response: This is an excellent question. It is possible. We have puzzled on this question for a long time. We have proven that the mutation in two alleles causes a translation stop codon removing 29% of the C-terminus of C1-FDX, and this protein loses its protein-protein interaction capability at least in the Y2H assay. Yet, the mutants are not severely affected, especially in later development. Hence, the presence of CI and CV implies they can be assembled, and only during seed germination it depends more on C1-FDX. Whether another mFDX or other proteins have a redundant function is something to look for.

**Reviewer #2:**

Chen and co-workers present a study to elucidate the function of a mitochondrial ferredoxin, C1-FDX, which is located in the so-called bridge module of mitochondrial complex I in plants, alga and Tetrahymena. The bridge module links the carbonic anhydrase module with the ubiquinone-reduction domain of complex I. Using CRISPR-Cas9 methodology, two Arabidopsis C1-FDX mutants were generated. Both carry a point mutation causing a shortened open reading frame. Mutant plants show delayed germination and decreased levels of complex I and complex V, as shown by Blue-native (BN) PAGE in combination with in-gel activity measurements. In contrast, overexpression of C1-FDX leads to increased levels of complexes I and V. Finally, abundances of different subunits of the complexes I and V are investigated in wildtype and mutant strains, as well as protein-protein interactions using Y2H, bimolecular fluorescence complementation (BiFC) and co-immunoprecipitation assays (Co-IP). The authors conclude that C1-FDX directly interacts with complexes I and V and is important for the assembly of both complexes. The authors present a wide range of experiments. The presented results are interesting, but nevertheless I have a few questions and objections:

Figure 1: Is C1-FDX absent in the two CRISPR-Cas9-lines c1-fdx-1 and c1-fdx-2? Since the mutation is at position 338, truncated proteins of 113 amino acids (instead of 159 amino acids) could be translated and imported into the mitochondria (in the truncated proteins, the C-terminal 46 amino acids would be absent). The truncated genes are transcribed (Fig. S3C). Presence or absence of C1-FDX could be clarified by MS/MS analyses of complex I bands from a BN gel.

Response: Yes. The c1-fdx-1 and c1-fdx-2 mutants can potentially be transcribed and translated into a truncated c1-fdx, losing 46 amino acid residues of its C-terminus. Whether this truncated protein is functional is questionable. We did an MS/MS analysis of CI in the WT and the mutant. We detected three C1-FDX peptides in the WT and none in the c1-fdx mutant. We also performed the LC-MS/MS analysis on the F’ complex with an increased detection time to test whether this truncated protein can still be incorporated into the F’ complex. The analysis detected five C1-FDX peptides (coverage 25.56%) in the F’ complex in the wildtype and the complemented c1-fdx-1 mutant but none in the c1-fdx-1 mutant (Table S1). This result indicates that the truncated protein loses its capability to incorporate into the F’ complex. We also tested the truncated c1-fdx protein for its interaction with the proteins that C1-FDX showed interaction in Y2H. The truncated protein lost the capability to interact with all the proteins (Figure 5 and S10). These data suggested that even when the truncated c1-fdx is synthesized, it lost some of its function at least. These data were added in Table S1 and Figure S10 in the revised manuscript.

A corresponding experiment actually has been done and is presented in the end of the manuscript (Table S1): The F’-band of complex V from wildtype (wt)-plants and from the mutants c1-fdx-1 & c1-fdx-1::C1-FDX-GFP was cut out from a BN gel and analyzed by MS/MS. C1-FDX was found in wt plants but not in mutant c1-fdx-1 (please add the amino acid sequences of the identified peptides in table S1; please present a complete list of all identified proteins). However, in wt plants, only one single peptide of C1-FDX was detected (the protein coverage is only 5.7%). Unfortunately, presence of C1-FDX is therefore not well secured. C1-FDX is a hydrophilic protein and previously several peptides have been detected by MS/MS, e.g. 9 peptides in Klusch et al. 2023 (Supp. table 4; the resulting protein coverage is 60%). The low level of C1-FDX detection in wt plants could be interpreted as a C1-FDX background on the BN gel in the region of the F’-band of complex V.

Response: Thank you for the suggestion. We provided all the protein data that we detected in Table S1. We agree that one CI-FDX peptide detected is not convincing. Following your suggestion, we performed LC-MS/MS analysis again, as mentioned above. This time, we increased the sample amount in the LC-MS/MS analysis for the WT, complement line, and c1-fdx mutant. Five peptides of C1-FDX with a coverage of 29.56% were detected in the wildtype, while no peptide of C1-FDX was detected in the *c1-fdx-1*, indicating that the truncated c1-fdx lost the capability to incorporate into F’. We noticed that the peptide number of C1-FDX and its coverage in our analyses is lower than that reported by Klusch et al. (2023). This might be because Klusch et al. used the supercomplex (I+III_2_) containing fractions as the sample for LC-MS/MS analysis, which greatly reduced the interfering proteins.

In the future (not for the current manuscript), complexome profiling experiments of mitochondria from wt-plants and C1-FDX mutant plants should be carried out to systematically characterize C1-FDX-containing assembly intermediates of the complexes I and V.

Response: Thanks for your advice. Complexome profiling experiments of mitochondria complex is a good approach to characterize their assembly. We will use this methodology in future studies.

Figure 2 and 3: Please indicate the protein amounts loaded per lane of the gels and that equal protein amounts were always loaded for wt and the mutants, respectively.

Response: According to your suggestion, we added the protein amounts loaded per lane for wildtype and *c1-fdx-1* in the legend of Figure 2: “The mitochondria were solubilized with dodecylmaltoside, and 130 μg mitochondrial proteins from wildtype and *c1-fdx1-1* were resolved in 3.5-12% BN-PAGE and assayed for activity (refer to methods)”.

Figures 2-4: I can follow that absence or truncation of C1-FDX in c1-fdx-1 and c1-fdx-2 mutant lines affects abundance and activity of complex I and complex V. For complex I, this has been shown previously using two mutant C1-FDX Arabidopsis-lines obtained by the CRISPR-Cas9 methodology (Röhrich et al. 2023). In these mutants, which seem to be true deletions, complex V was not affected. The discrepancy of these results and the results obtained by the analysis of the c1-fdx-1 and c1-fdx-2 lines should be discussed.

Response: Yes, the abundance of complex V was not observed differently between wildtype and the C1-FDX mutants in Röhrich’s data (Röhrich et al. 2023) as well as our data (Figure 2A). Because the decrease in the abundance of the α subunit is similar to the increase in the abundance of the β subunit, the loss function of C1-FDX mainly affected the assembly of F and F’. We also stated this phenomenon in the results in Line 215-216: “In addition, no change at the position of CV was detected between the wildtype and the *c1-fdx-1* mutant using the subunit *a* antibody (Figure 3B) and Coomassie brilliant blue staining (Figure 3A, Röhrich et al. 2023). These results indicate that the mutation of C1-FDX primarily affects the F1 module assembly.”

Could complex V reduction in the c1-fdx-1 and c1-fdx-2 mutants be based on an indirect effect? Could it be that reduction in complex I in the c1-fdx-1 and c1-fdx-2 mutants results in a decreased proton gradient across the inner mitochondrial membrane, which causes downregulation of genes encoding subunits of the mitochondrial ATP synthase complex, which uses the proton gradient for ATP synthesis? Please comment. On the other hand, the authors show direct interaction of C1-FDX and other subunits of the complexes I as well as subunits of complex V by Y2H, BiFC and Co-IP (Figure 5). Some of the results are nicely in line with published structural data for Arabidopsis complex I, some others are surprising. I am not an expert on evaluating these results. Can false-positive results be excluded using these procedures? Please comment.

Response:

(1) Although we cannot completely exclude the possibility that complex V reduction in the *c1-fdx-1* and *c1-fdx-2* mutants could be an indirect effect, it is highly unlikely. It is observed in various defective CI mutants where impaired CI does not affect the abundance or activity of CV or even increases the CV abundance and activity in these mutants, such as *tim23-2* (Wang et al., 2012), *ca2* and *ca1/ca2* (Fromm et al., 2016), *ciaf1* (Ivanova et al., 2019).

(2) To exclude the false-positive of the interaction data, we added the negative control of BiFC and the truncated c1-fdx-1 interaction results in Figure S10 and S11.

Reference:

Fromm, S., Braun, H. P., and Peterhansel, C. (2016). Mitochondrial gamma carbonic anhydrases are required for complex I assembly and plant reproductive development. New Phytologist, 211(1), 194-207.

Ivanova, A., Gill-Hille, M., Huang, S., Branca, R.M., Kmiec, B., Teixeira, P.F., Lehtiö, J., Whelan, J., and Murcha, M.W. (2019). A mitochondrial LYR protein is required for complex I assembly. Plant Physiology, 181: 1632-1650.

Wang, Y., Carrie, C., Giraud, E., Elhafez, D., Narsai, R., Duncan, O., & Murcha, M. W. (2012). Dual location of the mitochondrial preprotein transporters B14. 7 and Tim23-2 in complex I and the TIM17: 23 complex in Arabidopsis links mitochondrial activity and biogenesis. The Plant Cell, 24(6), 2675-2695.

**Minor points:**
Introduction section, Line 48: “inter-outer membrane space”, rather write “intermembrane space”.

Response: Thanks, we changed the “inter-outer membrane space” into “intermembrane space” in Line 60.

Results section, lanes 156, 203, 214, 227, 269: please avoid “to verify…”, but rather use “to test…”, to demonstrate a neutral attitude with regard to your hypotheses.

Response: Thanks. We changed the statement of “to verify…” into “to test…” in Lines 169, 219, 230, 243, 283.

Discussion section: There is a new hypothesis on C1-FDX function which you might want to consider for discussion (PubMed 37599162).

Response: Thank you for your recommendation. This paper, “Promotion of oxidative phosphorylation by complex I-anchored carbonic anhydrases?” offers a new perspective on the function of the CA domain and ferredoxin bridge in complex I. We cited this paper in our revised manuscript in Lines 295-296: “The iron ion of C1-FDX may assist the proton migration toward the ND2 half-channel entrance by a positive repulsive electrostatic force (Braun et al., 2023). In this study, we found that C1-FDX is also involved in the assembly of CI*.”

**Reviewer #3:**

The manuscript “C1-FDX is required for the assembly of mitochondrial complexes I and V in Arabidopsis” describes the creation (using CRISPR-Cas9) and characterisation of Arabidopsis mutants impaired in the expression of C1-FDX, a component of a ferredoxin bridge known to be important for complex I assembly and supercomplex (I+III) formation.

The introduction is informative and well written, and leads to the question: what is the precise role of the C1-FDX protein in Arabidopsis? Experiments are well conducted, and the results mostly clearly shown, apart from the western blots on BN gels. This is a big issue because most of the conclusions on involvement of C1-FDX in assembly of Complex V are based on these experiments.

The main problem as far as I am concerned is in the discussion. Although the evidence in favour of C1-FDX acting as an assembly factor in complex I assembly is strong, I am less convinced about its role in Complex V assembly. The data from the BN PAGE gels and especially the western blot images are not easy to interpret as they often appear over-exposed, making the quantification of the bands rather unreliable.

The discussion concerning the pathways proposed is difficult to follow. It is not very clear probably because the evidence is not fully convincing and suffers from slight over-interpretation. Each stage of the assembly processes of both Complex I and Complex V should be described more clearly, referring to the model provided.

Major points:
- The signals on several western blot images in Figure 3A and Figure 4 D, E and F, including the short exposure images, seem saturated to me. If so, the quantification using Image J cannot be accurate.

Response: Yes, some western blot images in the Figures are probably saturated. Therefore, we did not use Image J to quantify them. We stopped using these bands quantitatively.

- For example, line 178, the authors claim that CI* was also decreased in the mutant (Figure 3A). Although it is obvious with the anti-Nad9 antibody, it is a lot less clear with the anti-A5 antibody, and CI* is not detected with the anti-V1 antibody (Figure 3A), which I would expect to recognize CI* as it has the N module attached to it. Can the authors comment on that?

Response: The image with the anti-A5 antibody in Figure 3A was probably over-exposed. Thus, we replaced it with an image of less exposure time. This image clearly showed the difference in the abundance of CI* between the wildtype and the mutant.

Yes. V1, a subunit of the N module, should be detected at the CI* position by western blotting using antibody V1. The V1 antibody can recognize the V1 subunit in CI but not so well in CI*, even with a long-time exposure (as shown in the following Figure A). However, this anti-V1 antibody recognized the V1 subunit at the CI* position in the maize mutant (accumulating CI*), suggesting that this antibody can detect V1 but is less sensitive. Thus, one possibility for V1 not detected in CI* may be caused by a low amount of V1 in the gel. The wildtype and c1-fdx mutant do not accumulate a large amount of CI*. Another possibility is that, somehow, the conformation of V1 in CI and CI* might be different, although they were denatured in the BN gel. Other than these speculations, we are short of a convincing answer.

.

- In Figure 4A, it is obvious from the Coomassie blue staining (which is proportional to the quantity of protein in each band) that the mitochondrial protein pattern of the complemented line is very different from that of the WT and the mutant. Some major bands are missing from the complemented line, which in turn has many additional bands. This suggests that the “equal” mitochondrial protein loadings measured by Bradford assay are not equivalent in all lines, and that comparing quantities of the different sub-complexes between lanes is difficult because of that. That, in addition to the saturated signals and the poor resolution of BN gels in general, makes the interpretation rather unconvincing.

Response: We noticed the difference in the protein banding pattern between the complemented line and the WT. To gain more information on these lines, we analyzed the mitochondrial proteins from four *c1-fdx-1:*:C1-FDX-GFP lines and compared them with the WT. As shown in the following figure, the four complemented lines displayed a similar protein pattern, but all different from that in the wildtype. This result is similar to that in Figure 4A. It is possible that the different mitochondrial protein pattern between the transgenic lines and the wildtype is caused by over-expression of C1-FDX-GFP. The protein quantification is reliable, but the protein composition might be different between the complemented lines and the wildtype. It reflects, to some extent, the impact on mitochondrial proteins by over-expression of C1-FDX-GFP. We presented the data based on an equal amount of mitochondrial proteins.

- Line 235, the authors state: ‘In addition, the C1-FDX-GFP was also detected in the α/β sub-complex (Figure 4D and E). Compared with the wildtype, the abundance of α subunit in the α/β sub-complex was decreased in c1-fdx-1, while the abundance of β subunit was increased, suggesting that C1-FDX may play a role in the α/β assembly. This may be the cause for the altered α:β stoichiometry in c1-fdx-1 (Figure 3B). Therefore, C1-FDX plays a role in CI and CV assembly during seed germination in Arabidopsis.’

I do not agree with that statement, because in Fig 4 E, the mutant and the complemented line both have similar signals for the so called α:β intermediate complex, which seems to be mostly comprising the β subunit. As for the complemented line, I would have expected a similar signal for the α and β subunits westerns, as it expresses high levels of C1-APX, but this is not the case. There is still a lot less α subunit in the so called α:β intermediate complex, suggesting that C1-APX is not needed for assembly of the α:β sub-complex.

Therefore, the authors should be less assertive in their conclusions concerning the role of C1-APX in the assembly of Complex V.

Response: Thanks for your comments. Here is our interpretation of the results in Figures 4D and 4E. These are the same membrane hybridized with the anti-α and anti-β antibodies. Clearly, the anti-β antibody is more sensitive than the anti-α antibody in the detection. Compared with the wildtype α/β subcomplex that was barely detectable by the anti-α and anti-β antibodies, there is a substantially increased amount of β subunit in the α/β subcomplex (Figure 4E in the middle lane), while the α subunit was undetectable at the same position by the anti-α antibody even at a long exposure (Figure 4D, left image). This result indicates that the ratio between the α subunit and β subunit is altered in the c1-fdx mutant. Mutation of C1-FDX leads to more β subunits in the α/β subcomplex. We cannot compare two immune signals because antibody sensitivity can be different. However, we can gain information on the ratio changes in different samples. Our description of this paragraph is not clear, and we also over-interpret the result by claiming that the data suggest that C1-FDX may be involved in α/β subcomplex assembly. We removed this sentence.

Taking together all the results, we think the conclusion that C1-FDX is involved in CV assembly is supported. Mutation of C1-FDX affects the α:β intermediate complex (Figure 4D and 4E). The activity of CV and F1 is reduced in the *c1-fdx-1* mutant (Figure 2A). The C1-FDX-GFP fusion protein was also accumulated at the F’ in the transgenic line that rescued the phenotype (Figure 4F). All these F’ subunits were verified at this position by LC-MS/MS (Table S1). Protein interaction analysis shows that C1-FDX, as well as ACP1/2, can interact with complex V subunits by Co-IP, Y2H, and BiFC assays (Figure 5). The physical association of C1-FDX with CV components and the impacts on CV and its subcomplex in the *c1-fdx* mutant propelled us to the conclusion that C1-FDX is involved in the assembly of CV. We are currently testing the relationship between C1-FDX and the CV assembly factors. One factor showed interaction in Y2H.

Minor points:
1. In Figure 3, The MW marker sizes are not indicated.

Response: We added the molecular marker in Figure 3.

2. In Figure 4G, the Complex V activity bands are very difficult to see, so it is difficult to assess if CV, F’ or F1 are increased.

Response: Yes, the bands of CV, F’, and F1 are a little bit fuzzy. In Figure 4, we run the 15 lanes of 4-16% blue native gel. This gel only loads half the amount of proteins and needs to run a long time compared to the 3.5-12% blue native gel. Therefore, the bands are slightly fuzzy. But it still showed that the activity of CV and F1 in the *c1-fdx-1:*: C1-FDX line is slightly higher than the wildtype.

3. The blue and black arrows in Figure 6 are not very easy to tell apart. I would suggest using different shapes for arrows showing the various steps in the assembly model (black arrows) and to indicate abundance variations (red and blue arrows).

Role of C1-APX in the assembly

Response: Thank you for the suggestion. We changed the shape of the block arrows in Figure 6, which is easy to distinguish from the red and blue arrows.

4. In conclusion, I agree with the conclusions that C1-APX is involved in the assembly of Complex I because the evidence is strong, but I am more doubtful about what its involvement in Complex V is.

I think the results and discussion concerning this aspect needs to be rewritten if the authors present better western blots and want too fully convince the reader.

The manuscript should be improved in order to be convincing on this point or the authors should be less affirmative about the role of C1-FDX in Complex V assembly.

Response: Thanks for your suggestion. We rewrote these parts. We moved the section “C1-FDX is involved in the assembly of CI* in Arabidopsis” in the discussion to the first part. In addition, we weakened our statement that C1-FDX is involved in the assembly of complex V in the discussion. We changed the subtitle “C1-FDX functions an assembly factor in the assembly of CV in Arabidopsis” to “C1-FDX might be an assembly factor in the assembly of CV in Arabidopsis”.
