# Supplementary material for: C1-FDX is required for the assembly of mitochondrial complex I and subcomplexes of complex V in Arabidopsis
Source: PLoS Genet. 2024 Oct 2;20(10):e1011419. doi: 10.1371/journal.pgen.1011419 (PMC11446459; doi:10.1371/journal.pgen.1011419)
Supplement: Attachment — Submitted filename: Response to the reviewers_C1-FDX_PLOS genetics_R2.docx [file pgen.1011419.s016.docx]

**Reviewer #1:**

Considering the revised version of the manuscript entitled “C1-FDX is required for the assembly of mitochondrial complexes I and V in Arabidopsis” by Baoyin Chen et al., I find that the authors have adequately addressed most of my comments.
Only two comments, in my previous point 4.

4-The manuscript mentions that overexpressed C1-FDX-GFP leads to increased amounts of complex I and activity. An explanation for this phenomenon should be provided. I consider that the response was speculative and I think that the authors should reconsider the idea of increased amount of CI in the complemented plants with CI-FDX.

Response: Thanks for your suggestion. We detected a dramatic increase in the amount and activity of CI* and a slight increase of CI in the C1-FDX-GFP over-expression lines based on the quantification of the bands in Figure 4B. As the increase in CI is not as significant as CI*, we focused on CI* in the result description and discussion. The increased CI* is caused by over-expressing C1-FDX-GFP. One possibility is that C1-FDX promotes the assembly of CI*; thus, over-expression leads to an accumulation of CI*. This possibility is consistent with the fact that the loss of C1-FDX significantly reduces the CI and CI*, indicating that C1-FDX is required for the assembly of CI and CI*. Another possibility is that C1-FDX-GFP over-expression may reduce the assembly efficiency of CI* with the P_D_ module, resulting in an accumulation of CI*. In each case, C1-FDX is required for CI assembly. In contrast to the CI amount and activity, over-expression of C1-FDX-GFP significantly increased the amount and activity of CV as detected by the activity and protein blotting analysis.

And in my previous point 5,

5-Concerning interaction experiments, the lack of interaction of CAL2 with any other protein raises questions. Clarification on the physiological significance of this observation is warranted, particularly considering the interchangeability of CAL1 and CAL2 in CI models. I find this result to be strange and believe it should be interpreted with some caution.

Response: We also find it strange, considering the interchangeability of CAL1 and CAL2 in the CA2 and a 90% sequence identity between the two proteins. We checked the experimental procedures and the sequences of the constructs used in the Y2H experiment. We only used Y2H to test the interaction; somehow, CAL1 and CAL2 showed different interaction activities. The Y2H system may cause this difference. CA1, CA2, and CAL2 contact each other in the CI complex (Klusch et al., 2021, Plant Cell). However, CA1 and CA2 did not interact in the yeast two-hybrid assay (Córdoba et al., 2016).

We added the explanation in the revised manuscript in lines 275-280: ACP1 and ACP2 showed interaction only with CAL1. Considering the interchangeability of CAL1 and CAL2 in the CA domain and a 90% sequence identity between the two proteins (Wang et al., 2012), the different behavior of CAL1 and CAL2 may be due to some unknown differences when expressed in yeasts. It is known that CA1, CA2, and CAL2 contact each other in the CI complex (Klusch et al., 2021). However, CA1 and CA2 did not interact in the yeast two-hybrid assay (Córdoba et al., 2016).

Reference

Córdoba J P, Marchetti F, Soto D, et al. The CA domain of the respiratory complex I is required for normal embryogenesis in *Arabidopsis thalian*a. Journal of Experimental Botany, 2016, 67(5): 1589-1603.

Klusch N, Senkler J, Yildiz Ö, Kühlbrandt W, Braun HP. A ferredoxin bridge connects the two arms of plant mitochondrial complex I. Plant Cell. 2021. 33: 2072-2091.

Wang Q, Fristedt R, Yu X, et al. The γ-carbonic anhydrase subcomplex of mitochondrial complex I is essential for development and important for photomorphogenesis of Arabidopsis. Plant Physiology, 2012, 160(3): 1373-1383.

**Reviewer #2:**

From reading the answers of the authors to the concerns of the reviewers I understand that the role of C1-FDX in complex I assembly is obvious but the role of C1-FDX in complex V assembly is less clear. Very last statement of the authors: „… we weakened our statement that C1-FDX is involved in the assembly of complex V in the discussion”. However, this should not only be weakened in the discussion but also in the title and in the abstract of the manuscript. Title of the revised version: „C1-FDX is required for the assembly of mitochondrial complexes I and V in Arabidopsis“. Abstract of the revised version: „Molecular analyses showed that loss of the C1-FDX function decreases the abundance and activity of both CI and CV.“ In my previous review, I pointed to the study of Röhrich et al. 2023, who also characterized two mutant Arabidopsis-lines deficient in C1-FDX. In these mutants, which seem to be true deletions, complex V abundance was not affected. I asked the authors to discuss the discrepancy of these results and the results obtained for c1-fdx-1 and c1-fdx-2. Answer of the authors: „Yes, the abundance of complex V was not observed differently between wildtype and the C1-FDX mutants in Röhrich’s data (Röhrich et al. 2023) as well as our data (Figure 2A). Because the decrease in the abundance of the α subunit is similar to the increase in the abundance of the β subunit, the loss function of C1-FDX mainly affected the assembly of F and F’.“ If I understand this statement correctly, the title of the manuscript rather should be changed into „C1-FDX is required for the assembly of mitochondrial complex I as well as subcomplexes of complex V in Arabidopsis“. Similarly, in the abstract section, it should be stated that the C1-FDX absence affects the abundance of complex I and subcomplexes of complex V.

Response: Thanks for your suggestion. We have changed the title to “C1-FDX is required for the assembly of mitochondrial complex I and subcomplexes of complex V in Arabidopsis”. In the abstract, we also stated that “Molecular analyses showed that loss of the C1-FDX function decreases the abundance and activity of both CI and subcomplexes of CV” in lines 35-36. In the discussion, we also stated that “C1-FDX may promote the subcomplexes assembly of CV in Arabidopsis” in line 338, “In this study, we show that C1-FDX not only serves as a structural component of CI but also possesses the function of an assembly factor in the subcomplexes assembly of CV during seed germination in Arabidopsis mitochondria.” in lines 341-343, and “Taken together, the physical interaction with the CV subunits, the association with the F’ and F1 sub-complexes, and the genetic data demonstrate that C1-FDX is involved in the subcomplexes V assembly process.” in lines 353-356.

By the way, “because the decrease in the abundance of the α subunit is similar to the increase in the abundance of the β subunit, the loss function of C1-FDX mainly affected the assembly of F and F’.” – do you mean that beta-subunits might substitute alpha subunits in the holo complex V? Considering the published structures of complex V, this would be highly unlikely.

Response: We noticed this sentence is misleading. Thanks for pointing it out. We mean that an increase in the β subunit abundance accompanied the decrease in the α subunit abundance, suggesting that the loss function of C1-FDX mainly affected the assembly of F and F’. It was changed in the text.

We do not know the conformation of the altered ratio α and β subunits in the subcomplexes. The α and β subunits can form α-α and β-β homodimers (Wang and Ackerman, 2000; Wang et al., 2000), raising the possibility that an aberrant α/β complex might be formed.

**Reviewer #3:**

Thank you for addressing the comments and toning down your conclusions in this new version of the manuscript, although I am still not fully convinced by the conclusions about the role of C1-FDX in complex V assembly.

Response: Thank you for allowing the tuned-down conclusion on the CV assembly. Considering the suggestion by reviewer 2, we no longer insist on concluding that C1-FDX is required for the assembly of complex V. Instead, we tuned down to the conclusion that C1-FDX is required for the assembly of complex V subcomplexes in Arabidopsis, as reflected in the title, abstract, and discussion.

Just a note, BN (Blue native PAGE) is by definition non-denaturing. It uses mild detergents that isolate the protein complexes from the membranes.

Response: Thanks for the reminder. We will pay attention to our wording in the statement of Blue Native PAGE.
